# The Professional Agency in Supervision of General Practitioners Before Their Supervisor Training Module: A Narrative Positioning Analysis

**DOI:** 10.1097/CEH.0000000000000596

**Published:** 2025-02-05

**Authors:** Katri Salokangas, Sanna Vehviläinen, Nina Tusa, Anita Malinen, Pekka Mäntyselkä

**Affiliations:** **Mrs. Salokangas:** Doctoral researcher, Institute of Public Health and Clinical Nutrition, University of Eastern Finland, Kuopio, Finland.; **Prof. Vehviläinen:** Professor of career counseling, School of Educational Sciences and Psychology, University of Eastern Finland, Joensuu, Finland.; **Dr. Tusa:** Chief physician of education, Wellbeing Services County of North Savo, Educational Services, Kuopio, Finland, and Institute of Public Health and Clinical Nutrition, University of Eastern Finland, Kuopio, Finland.; **Mrs. Malinen:** Lecturer, Department of Education, University of Jyväskylä, Jyväskylä, Finland.; **Prof. Mäntyselkä:** Professor of general practice, Institute of Public Health and Clinical Nutrition, University of Eastern Finland, Kuopio, Finland, and Clinical Research and Trials Centre, Kuopio University Hospital, Wellbeing Services County of North Savo, Kuopio, Finland.

**Keywords:** general practice, supervising, supervisor training, professional agency, medical continuing vocational education, competence-based medical education

## Abstract

**Introduction:**

Supervising in the workplace plays a key role in increasing the skills of the trainee. The shift to competence-based medical education requires both clinical expertise and pedagogical skills from the supervisor. These are distinct types of expertise. We know only a little of how competencies of supervising develop in medical education. The aim of our study is to find out what kind of professional agency in supervision general practitioners describes before their supervisor training. Professional agency refers to the individual's skills, willingness, ability, and responsibility to act with others in the professional context.

**Methods:**

Our participants wrote a presentation of themselves before starting a supervisor training module. We studied these texts with narrative positioning analysis to examine who they are as supervisors, that is, the kind of professional agency they describe.

**Results:**

We found three types of descriptions of professional agency: traditional master-apprenticeship supervision, clinical skills supervising with a collegial relationship, and process-oriented dialogical supervision. Supervising is mostly described as supervising the trainee on clinical skills and participants have a will to be a good supervisor, but they also express uncertainty of achieving this goal.

**Conclusion:**

The variation in general practice supervisors' agency and pedagogical skills poses challenges for training providers in how to tailor the training to suit the best to participants with different skills. However, it gives an excellent opportunity for fruitful peer-to-peer learning. With our findings, it is possible to further develop supervisor training.

Supervising in the workplace plays a key role in increasing the skills of the trainee^1,2^ and is an essential part of competency-based medical education (CBME). To carry out high-quality specialization training, supervisors need clinical expertise and pedagogical skills, especially skills to carry out supervision practices intertwined with daily clinical work tasks.^3–5^ Clinical competence and pedagogical skills are distinct kinds of expertise, and pedagogical skills do not develop without pedagogical training. Even though physicians have always taught the younger ones, every doctor is not automatically an educator.^6^ Pedagogical skills are not a part of the core clinical competencies of the profession, and their development should not be taken for granted.^6^ So far, we know very little of how supervisory competencies develop in the education of health professionals.^7^ The requirements for supervisors' skills have increased but there is still very little research on how supervisors strengthen their pedagogical and supervising competency.^4,8^ Supervisors need support from their superiors and work community, training, and supportive concrete practices at the workplace.^9^ The transition to CBME has been prompt, but its implementation in working life has not been systematic and there is still a lack of shared practices.^5^ Therefore, some models for systematizing supervision and supporting supervisors have been developed in recent years.^8,10,11^ These models describe different supervisory roles and styles that the supervisor should manage and be able to use, as well as ways to reinforce the supervisor's identity as educator, for example, by creating peer-to-peer networks.^8,10,11^

In the Finnish medical specialization system, the switch to CBME started in 2020,^12^ and the training takes 5 to 6 years.^13^ In Finland, trainees work as physicians responsible for treatment and meet patients independently under supervision. For example, general practice trainees work in their own rooms and have their own patient list from the normal patient flow. In Finland, general practitioners (GPs) treat patients of all ages, take care of preventive work, and diagnose and treat diseases from simple to combinations. GPs can consult the hospital specialist, but the coordinating responsibility stays in primary care.^14^ The amount of time for supervision is defined in work schedules (4 h/mo), in addition to consultations taking place alongside clinical work. Trainees have basically the same responsibilities as the more experienced physicians, thus specialists and trainees are relatively equal. According to national surveys in health centers, only approximately 30% of physicians are GP specialists and approximately 70% are doctors in the training phase or are nonspecialized.^15^ In view of the above, it is important to support the supervisory skills of GP supervisors and to further develop the culture of supervision in primary health care.

In the study of professional expertise and workplace learning, there is a growing interest in the so-called professional agency of experts.^16–21^ Professional agency refers to the possibilities for action and participation, provided by the various affordances of a social context. Agency is mediated by both social structures (such as work practices, norms, resources, and leadership), daily interactions, and working cultures, and individual goals, skills, and learning.^16–21^ Thus, professional agency is constructed by both the individual and the social environment. Individuals have professional agency to the extent they are able and willing to act, take a stand, and develop practices in their work. To do this, they also rely on their colleagues' contributions and skills. Strong agency has been found to enable development in expertise, well-being, and resilience.^16–21^ Prior research has focused on supervisory styles and roles.^11,22,23^ However, in skilled supervision, should interaction styles vary according to the situation and changes in the learners' skill. Therefore, we chose the perspective of professional agency. It is broader concept than “skill” or “expertise,” covering, among other things, the ability to vary supervisory styles and roles. Furthermore, the concept of agency incorporates the idea that an individual's possibility to act is always shaped by, not only the individual's skills and motivation, but also the extent to which the social environment enables it. For instance, when the professional agency of the supervisor is strong, he/she can use different styles and adjust his/her role according to the needs and situations of the trainee and according to the affordances provided by the local learning environment. In our case, the strong agency of the supervisors means that they can act in accordance with the supervisory models built for the needs of CBME and use various supervisory styles that support the strengthening of the trainee's competence in changing situations. Strong professional agency also means that the supervisor is willing to facilitate the actions of others at the workplace.

Recent research in medical education has pointed out that more research should be done on training and its planning in working life because classroom-based faculty development cannot ensure transfer of skills and knowledge into the workplace.^4,24^ Our institutions have been organizing a special training module for the supervisors since 2013. It is intended for GPs who supervise GP trainees in health centers, that is, in practical working life. In this substudy, we examine the manifestations of the professional agency in supervision of GPs who participated in this training module before the start of the training. Understanding the starting points for the learning process is essential for the training development. The results can be used to further develop supervisor training so that it best supports the needs of supervisors, trainees, working life, and patients. In addition, the culture of education in primary health care can be strengthened. The professional agency in supervision may, at best, also support the ability of the work community or organization to renew itself.^25^

## METHODS

The material of our study consists of the first written assignments of the participants in the supervisor training modules of 2018 to 2019 (N = 25) and 2020 to 2021 (N = 19). The module lasts 1 year and consists of three 2-day, face-to-face segments between which the participants supervise their trainees in their workplace, collect feedback, and reflect on the development of their supervision in written assignments. The contents of the face-to-face days include adult learning, CBME in GP training, dialogue skills in supervision, planning, and implementation of supervision, one's own supervisor identity, and feedback. The first written assignment is called “Pre-assignment 1.” The instructions of the task are “Now you can start the course by introducing yourself to your group. Tell the group about yourself: What kind of supervising did you receive during your specialization, and what kind of supervisor do you consider yourself? What kind of successes and challenges have you faced as a supervisor? What expectations and goals do you have for your beginning training?”

Participants completed this task before coming to the starting days of the module. In total, 44 (85%) course participants took part in our research. The participants were GPs, most of whom graduated as specialists in 2015 to 2018 (N = 30, 68%). About three-quarters (N = 32, 73%) of the participants were women.

The material was collected with the voluntary written permission of the participants, and there were no sanctions for nonparticipation. Written assignments were done during the module, so participation in the research has not caused any additional work. The material was pseudonymized and the results are presented in such a way that it is not possible to identify an individual participant. Owing to the above justifications, in accordance with the ethical review system for research in Finland^26^ and based on the response from the research ethics committee of our university, an ethical review was not required for this study.

The method chosen for the analysis is narrative positioning analysis^27,28^ that has been developed as an analytical method specifically for short fragments of stories, interview clips, or texts. It is used to determine the agencies of the storytellers by analyzing what kinds of positions they build in relation to other characters in the story, the listeners in the story, and the expectations, requirements, and dominant models associated with the phenomenon being acted on. By studying these positions, we can learn who the storytellers are in the narrative at hand, that is, what kind of agency they describe in the context and situations of the phenomenon. In our study, this means what kind of professional agency in supervising do our participants describe. The method is suitable for analyzing our data, because the background theories of our research, professional agency, and supervision are also relational concepts. Professional agency develops in relation to other agents and the work environment. Supervision is an activity between the supervisor and the trainee, and between the work community, the supervisor, and the trainee. In addition, supervision activities are in relation to the shared core task, that is, the work of a physician. Our material is also suitable for this method because the average length of the assignments is one A4 sheet.

At first, the first and the second authors read the material independently collecting answers to our research question: What kind of professional agency in supervision do the participants describe before their supervisor training? How our participants describe their supervisory skills and actions, their motivation to supervise and develop in it, their experiences in supervising, the obstacles, and enablers of their context in supervision, and cooperation with other agencies of supervision. Seven partly overlapping themes were found. The whole research group went through these themes together, discussing which ones were relevant and subject to further analysis. After that, the first author expanded on the analysis of these themes with the help of narrative positioning analysis and reflected them against theories of professional agency and supervisory agency. The first author regrouped the findings into three main categories and their subcategories according to the type of agency they exhibited and organized the data into a chart with descriptive quotations. Next, we present the findings showing how our study participants position themselves in relation to their trainees and peers, the providers of the training, and the supervision activity itself. This describes who they are as supervisors, that is, what kind of professional agency they express.

## RESULTS

We found three types of professional agencies in supervision: (1) traditional master-apprenticeship supervision, (2) supervising of clinical skills with a collegial relationship, and (3) process-oriented dialogical supervision. Each of these types have three descriptive dimensions summarized in Table 1.

**TABLE 1.** Comparison of the Dimensions of the Main Types of the Professional Agency in Supervision

Dimensions	Main Types		
	Traditional Master-Apprenticeship Supervision	Clinical Skills Supervising, Collegial Relationship	Process-Oriented Dialogical Supervision
The relation to trainees	Supervision depends on the trainee's skills and motivation—there is very little that the supervisor can do if the trainee does not act as expected There is a power structure: Supervisor as more experienced is in a higher position	Supervisors want to be on the same side as their trainees, but it is challenging for the supervisor that the trainees are different Supervisor and trainee are quite equal—both are benefitting	The learning process differs from one trainee to another—the supervisor must have versatile skills to meet the needs of the trainee There is continuous dialogue between the trainee and the supervisor
The relation to the need for peer support/mentorship	There is no reflection on the other participants in the supervising	Supervising is up to an individual supervisor Peer support is needed for supervisory practices and techniques	Peers, work community, and training providers are all needed to contribute to supervising
The relation to the need for supervisory development	It is enough that the supervisor has medical and clinical expertise—these are the unproblematic basis for pedagogy Knowledge is transferred to the trainee through model learning	There is a will to become a good supervisor, but supervisors have feelings of inadequacy: they have the skills of a specialist, but they have doubts whether that is enough	Supervision is focused on the learning process where the trainee becomes a specialist—the supervisor needs pedagogical skills to support the trainee’s own thinking and learning

### Traditional Master-Apprenticeship Supervision

In five (11%) writings, the supervising is presented in a manner of traditional master-apprenticeship. The supervisor is a role model showing how things are done, and the trainee acts as expected. There is a clear power structure between supervisors and trainees, with supervisors being in a higher position because they are more experienced. Trainees are expected to settle into their own position. Seniors have always trained juniors and there is no need for special supervising skills. Here supervising seems to be a transfer of the knowledge of the senior to the junior and there is no doubt whether that is enough or suitable for the trainee and his/her specialization path.

#### It Depends on the Trainee's Skills and Motivation Whether Supervision Works or Not

The results of supervision are based on what kind of persons trainees are. If they are motivated and want to learn and work, supervision works. But if they do not act the way the supervisor wants or expects, it seems that there is very little that the supervisor can do.

“It is a pleasure to look at an enthusiastic student. If, on the other hand, one’s attitude is negative or the starting expectation is unrealistic, it is more challenging to put together a successful period.” (ID1010)

#### It Is Enough That the Supervisor Has Clinical Skills

In this type, pedagogical skills are seen as somewhat unnecessary because more experienced physicians have always educated younger ones, and the medical and clinical expertise are treated as the unproblematic basis for pedagogy.

“My own supervisor has not been trained as a supervisor, so he did not supervise the way that is required today. But I think he has been successful in supervising.” (ID1035)

“I have not had any official supervising rights. Well, despite this, for years I have tutored young colleagues more or less systematically.” (ID1045)

#### Knowledge Is Transferred to the Trainee Through Model Learning

The supervisor is a master, so he/she knows how things are done and shows them to the trainee and that is the way trainees are educated. Supervisor teaches what he/she knows. It is rewarding for the supervisor to see trainees act in the way the supervisor has shown.

“It is great if I can share what I have learned to others. It is best if you have been able to get a young colleague excited by your own example or actions!” (ID1022)

### Clinical Skills Supervising, Collegial Relationship

In our data, supervising is mostly (68%) described as supervising a trainee in clinical skills and it is up to an individual supervisor. Here the supervisor and trainee are more equal than in the previous type, and both parties are seen to benefit. Participants present characterizations of a good supervisor, and they have a desire to become one. Unlike in the previous type, they express uncertainty and doubt, implying they still have something to learn as supervisors.

#### Desire to Be on the Side of the Trainee Even Though Diversity Poses Challenges

The participants describe a strong desire to be on the same side as their trainees. They want their trainees to know that they are there for them. The supervisory relationship is presented as relatively equal.

“As a supervisor, I think I am easily approachable, preferring to treat the trainee as an equal colleague.” (ID1006)

“I have tried to think primarily of what is best for the trainee.” (ID1035)

The participants contend that trainees are different in many ways. This is challenging for the supervisor because they feel their supervisory skills are limited.

“On one hand, the most challenging aspect of supervising is situations in which trainees feel that they already know a lot and do not feel that they need supervising. How to wake them up to understand that you are never ready in a physician’s job. On the other hand, situations in which trainees are insecure as a physician are also challenging. How can the growth and development of a young physician be supported?” (ID1003)

#### Peer Support Is Needed for Supervisory Practices and Techniques

Here, good supervising is described as having established a system of designated supervisors, a regularly recurring time for supervising in the schedules, and an agreed topic and content for the supervising. In general, the topics consist of various patient cases and clinical problems or house practices. The need for peer support is mainly described as an exchange of ideas on the means of implementing these technical matters.

“I look forward to meeting with other supervisors and discussing how supervision has been implemented in their workplaces and what kind of practical supervisory methods they have.” (ID1039)

#### Desire to Perform Good Supervising: The Skills of a Specialist Are There, But Are They Enough?

Our participants shared their experiences of good supervision, and many were able to name a particularly skillful supervisor from their own specialization path. Inspired by these, they too want to become good supervisors.

“I have had great supervision, the supervisor has listened to the professional matters and a bit to the side, the discussion has been open, the conflicts constructive and in situations only the ‘things’ have quarrelled, never the persons.” (ID1009)

All participants are GPs and many of them have been a specialist for several years, so they do not have any problems in supervising trainees. But there were some doubts about whether that is enough: 16 (36%) participants expressed feelings of inadequacy.

“As a supervisor, I feel like a novice. In a short period of time, I have developed a way of being a supervisor that may not be the best possible. In supervising situations, the focus is clearly on patient cases. Growing up in the profession is a challenge.” (ID1008)

### Process-Oriented Dialogical Supervision

In nine (20%) writings, the supervising is presented as focusing on the learning process in which the trainee becomes a GP. The path is different with every trainee and the supervisor's task is to understand it and find ways to support the trainee along the way. The relationship involves continuous dialogue between the supervisor and the trainee. This is a very different view from the master-apprenticeship type where the supervisor transfers his/her own knowledge to the trainee without any questioning. Another importantly different feature compared with the previous more restricted supervisory types is, that the supervisor is willing and able to use the help of the entire work community in supervising, it is not the responsibility of the supervisor alone.

#### Trainees Are Allowed to Be Different: Supervisor Needs Varying Skills

In this type, the diversity of trainees is seen as a normativity and as a good thing. One can become a good general practitioner from very different backgrounds and through various paths. The supervisor must have versatile skills to meet the needs of the trainees.

“I sincerely believe that even with a wide variety of personalities and styles one can be a good general practitioner. There is room for diversity.” (ID1020)

“As a supervisor, I think the biggest challenges are finding the most suitable way of supervising individually. Not everyone learns the same way and an approach that works with one may not be the best for the other.” (ID1015)

#### Peers, Work Community, and Training Providers Are All Needed to Contribute to Supervising

In our data, there were six (14%) instances of the idea that there is a need for networking with peers to reflect on supervising and a wish to have the entire work community engaged in supervising. This is quite different from the main perspective of peer support being a channel to ask how others technically arrange supervision in their workplace.

“I expect peer-to-peer learning and new supervising insights from the training.” (ID1020)

“I am trying to find ways to involve other staff in supervising.” (ID1021)

All of this can be done with the help of the supervisor training providers.

“It is a relief to know that you can get help from the university if you need it.” (ID1035)

#### Supervision Is a Process: The Supervisor Is a Mentor and Needs Pedagogical Skills

In this type, supervision is referred to as mentoring, walking alongside trainees, supporting their own thinking, and learning. The supervision is focused on the learning process of the trainee, which is different for all trainees, and, therefore, the supervisor needs pedagogical skills such as understanding how adults learn and knowledge of different learning theories.

“The best thing about supervising is the process of a young, even insecure, doctor becoming a skilled, empathetic, and courageous physician.” (ID1003)

“As a supervisor, I am a mentor, I try to supervise in such a way that you do not give ready-made answers but instead help trainees to build their competence through the development of their own thinking.” (ID1020)

## DISCUSSION

In this study, we explored the narrations that are participants' personal perceptions of professional agency of GP supervisors before starting the supervisory training module. We found a variation of professional agency in their descriptions. The most restricted one is the master-apprenticeship type, where the supervisors view as their task to pass on their own knowledge to the trainees, and they treat this as the taken-for-granted basis for their expertise as supervisors. Here the supervisor training is not seen as necessary: the more experienced physicians have always taught the younger ones. Somewhat broader agency is described as clinical skills supervising with a collegial relationship, where the supervisors have strong specialist expertise and medical knowledge. However, they also experience doubt whether that is enough and a need for something more that seems to be pedagogical knowledge, and this is where new constructions of expertise can be reached with supervisor training. If the supervisors should stay in these restricted agencies, the needs of high-quality supervision in CBME cannot be reached. The broadest agency is described in process-oriented dialogical supervision, where supervisors can admit that they do not have to know everything, and the whole work community takes part in supervision. As the supervision focuses on the learning process, it best serves the different trainees in their various paths and situations, and needs. Furthermore, it enables building dialogical supervisory and work culture and hence strengthens the well-being of the whole work community. This variation should be considered by training providers when planning and developing training so that it meets the needs of different participants and provides fruitful challenges for participants with different supervisory agency.

Similar findings were made in a study of the academic supervision of PhD dissertations and master's theses.^29^ Supervisory agency is mainly described through the traditional model as the activity and responsibility of an individual supervisor, with different kinds of trainees posing challenges, and as the supervision of subject matter expertise. But there is an emerging idea of process orientation and of the communality of supervision, for example, from peer-to-peer activities. Here the self-efficacy may weaken because the supervisors realize they do not know everything, and this is the point where the training can support the supervisors to accept the uncertainty and learn a new interpretation of what expertise means. Similarities were also found in supervising trainees in hospital settings.^30^ There is a will to do what is best for the trainee but the differences among trainees pose challenges for the supervisor. Also, these supervisors expressed feelings of inadequacy whether they are good enough supervisors even though they are experienced physicians. This uncertainty can be harnessed in learning a new way of expertise in supervisor training and should be considered when tailoring the training.

A study investigating why doctors teach^31^ also made similar findings. Teaching was seen as part of being a doctor, as our participants described it “seniors have always educated the younger ones,” it is part of the work of a more experienced physician. The idea of “paying back for the education you yourself have received” was portrayed in our data as a desire to give back the good supervision you have received during specialization. These can be seen as incentives to become a supervisor and should be considered by the training providers how to strengthen the vision that being a supervisor is more than being a senior and that supervising may differ from what the participant has experienced.

Another hospital setting study focusing on physicians' self-perceived preparedness regarding supervision of medical students found that interaction between faculty and supervisors is crucial in supporting supervisors and increasing their confidence to supervise.^9^ Also, a support from the person who is responsible for supervising and meeting peer supervisors at the workplace is positively associated with preparedness to supervise.^9^ Similarities are also seen in our study: supervisors whose agency is broader have noticed that peers, work community, and training providers are all needed to contribute to supervising. Furthermore, the variation that we found in professional agency gives opportunities to diverse peer-to-peer learning and this should be encouraged by the training providers enabling participants to get to know each other and to build supervisor networks.

Our findings strengthen the perceptions of previous studies in both nonmedical and medical fields: supervising is depicted as work and duty of a more experienced professional and there is a will to be a good supervisor and do what is the best for the trainee. They also show that being a physician and being a supervisor are different kinds of expertise: even though all our study participants are specialized in general practice, they still have quite a limited agency in supervising. This is also supported by the findings from previous studies that the supervisor skills do not come automatically with the doctor skills^6^ or from own experiences of being supervised.^30^

To meet the requirements set by CBME,^3^ and be able to carry out the recently developed supervisory models,^8,10,11^ the supervisor must have strong agency. The professional agency of the supervisor expands and becomes more diversified when, in addition to acting as a traditional master-apprenticeship model and supervising the physician's clinical skills, there are skills to supervise the learning process, that is, the specialization path of the trainee and an understanding how to harness the competence of the whole work community for the supervision. To achieve this, it is important to train supervisors so that they learn this different way of perceiving expertise.

### Strengths and Weaknesses of Research

One main strength of the study is its working life affiliation, participants supervise trainees regularly in their work in health centers. With this study, we have responded the call for these kinds of studies.^4,24^

Our research team consists of physicians with pedagogical training and experience in the supervising of trainees in working life, and pedagogues with experience in adult education and supervising also in the medical continuing vocational training. Together we have a strong understanding of the study subject, and the various backgrounds have produced a diverse analysis of the material and multiple but consistent findings.

The assignment was in writing and the researcher did not meet the participants, so there were no opportunities to ask additional or clarifying questions. Participants returned their assignments under their own name so the training providers found out what each participant answered, and it could have affected what the participants wanted to write. In contrast, the participants were allowed to write quite freely, and the tasks had no acceptance requirements. Self-reports are subjective and may not be aligned with the actual teaching ability. We do not have access to their actual activities, so we cannot compare these self-reports with the true supervising practices. However, self-perceptions mediate the individual constructions of professional agency and thus they are valid data for our study. Participation in the study was voluntary, so it is possible that a person who does not like supervising would not participate and, therefore, negative aspects would remain unknown. However, most participants (85%) wanted to take part in the research. The participants have different backgrounds in terms of sex, age, motivation, and work experience, representing a diverse range of GP supervisors.

## CONCLUSION

In this study, we found a variation of professional agency from quite a restricted master-apprenticeship type to a significantly broader process-oriented dialogical type. Mostly the agency is described quite restricted where there are strong skills of a specialist and a good will to supervise but also some doubts about whether these are enough. Identifying these versatile self-perceptions of GP supervisors' skills helps to tailor the supervisor training so that it best suits to different participants and gives them more individualized support. Peer work is also found to be important in supervising and should, therefore, be encouraged by training providers. Furthermore, if most supervisors have restricted skills, they cannot use the supervisory styles that support the strengthening of the competency of the trainees,^11,23^ and cannot act the way that the newly developed supervisor models expect.^8,22^ That is why there is a need for supervisor training and our study findings can be used to further develop it.

**Lessons for Practice**

■ We found a variation in the descriptions of professional agency of general practice supervisors from quite restricted master-apprenticeship type to somewhat broader type of clinical skills supervising with a collegial relationship to the broadest one process-oriented dialogical supervision.
■ The variation should be considered by the training providers in how to tailor the training to suit the best to different participants and to give them more individualized support.
■ The variation gives an excellent opportunity to fruitful peer-to-peer learning and discussions, which should be encouraged by the training providers: enable supervisors to get to know each other and to build supervisor networks.
